# Psychopathology of schizophrenia in the context of the superior longitudinal fascicle integrity – a DTI study

**DOI:** 10.1192/j.eurpsy.2022.642

**Published:** 2022-09-01

**Authors:** P. Podwalski, E. Tyburski, K. Szczygieł, A. Michalczyk, L. Sagan, J. Samochowiec

**Affiliations:** 1 Pomeranian Medical University, Department Of Psychiatry, Szczecin, Poland; 2 Pomeranian Medical University,, Department Of Neurosurgery, Szczecin, Poland

**Keywords:** DTI, Neuroimaging, Psychopathology, schizophrénia

## Abstract

**Introduction:**

Schizophrenia is a chronic mental illness with unclear etiology. It is characterized by symptoms in various psychopathological domains (e.g. positive, negative). One of the concepts explaining the etiology of schizophrenia is the disconnection hypothesis. It assumes the existence of structural and functional disorders within the connections of brain regions. White matter is largely responsible for the quality of these connections. One of the important structures of white matter is the superior longitudinal fascicle (SLF) which connects many cortical structures.

**Objectives:**

The main aim of our study was to search for a relationship between the integrity of SLF and various psychopathological dimensions among schizophrenia patients.

**Methods:**

42 schizophrenia subjects (SS) and 32 healthy controls (HC) participated in the study. All study participants underwent DTI-MRI analysis. The psychopathology of SS was assessed using the Positive and Negative Syndrome Scale (PANSS). In the study, we used the PANSS dimensions proposed by Shafer. Then, the SLF analysis was performed using fractional anisotropy (FA) and mean diffusivity (MD) parameters.

**Results:**

The differences in FA and MD values in SLF bilaterally between groups were confirmed. A correlation was found between MD values in left SLF and positive symptoms (p = 0.040) and excitement (p = 0.012). A correlation was found between the MD values in the right SLF and the symptoms of disorganization (p <0.000) and excitement (p = 0.004).

**Conclusions:**

SLF seems to play a role in the etiopathogenesis of schizophrenia. Disturbed SLF integrity may be involved in the development of positive, disorganization and excitement symptoms.

**Disclosure:**

No significant relationships.

